# Identifying subpathway signatures for individualized anticancer drug response by integrating multi-omics data

**DOI:** 10.1186/s12967-019-2010-4

**Published:** 2019-08-06

**Authors:** Yanjun Xu, Qun Dong, Feng Li, Yingqi Xu, Congxue Hu, Jingwen Wang, Desi Shang, Xuan Zheng, Haixiu Yang, Chunlong Zhang, Mengting Shao, Mohan Meng, Zhiying Xiong, Xia Li, Yunpeng Zhang

**Affiliations:** College of Bioinformatics Science and Technology, Harbin Medical University, Harbin, 150081 China

**Keywords:** Subpathway signatures, Anticancer drug response, Multi-omics

## Abstract

**Background:**

Individualized drug response prediction is vital for achieving personalized treatment of cancer and moving precision medicine forward. Large-scale multi-omics profiles provide unprecedented opportunities for precision cancer therapy.

**Methods:**

In this study, we propose a pipeline to identify subpathway signatures for anticancer drug response of individuals by integrating the comprehensive contributions of multiple genetic and epigenetic (gene expression, copy number variation and DNA methylation) alterations.

**Results:**

Totally, 46 subpathway signatures associated with individual responses to different anticancer drugs were identified based on five cancer-drug response datasets. We have validated the reliability of subpathway signatures in two independent datasets. Furthermore, we also demonstrated these multi-omics subpathway signatures could significantly improve the performance of anticancer drug response prediction. In-depth analysis of these 46 subpathway signatures uncovered the essential roles of three omics types and the functional associations underlying different anticancer drug responses. Patient stratification based on subpathway signatures involved in anticancer drug response identified subtypes with different clinical outcomes, implying their potential roles as prognostic biomarkers. In addition, a landscape of subpathways associated with cellular responses to 191 anticancer drugs from CellMiner was provided and the mechanism similarity of drug action was accurately unclosed based on these subpathways. Finally, we constructed a user-friendly web interface-CancerDAP (http://bio-bigdata.hrbmu.edu.cn/CancerDAP/) available to explore 2751 subpathways relevant with 191 anticancer drugs response.

**Conclusions:**

Taken together, our study identified and systematically characterized subpathway signatures for individualized anticancer drug response prediction, which may promote the precise treatment of cancer and the study for molecular mechanisms of drug actions.

**Electronic supplementary material:**

The online version of this article (10.1186/s12967-019-2010-4) contains supplementary material, which is available to authorized users.

## Background

Due to the extensive genetic heterogeneity in human cancer, patients with seemingly the same tumor type always manifest widely variable responses to anticancer therapies [1–3]. Despite great effort to the development of cancer treatment, often these therapies are effective only in quite a few patients and the remaining will miss the best treatment time. One approach to settle this problem is to identify and apply molecular biomarkers to accurately predict anticancer drug response for individuals. The rapid advances and reduced costs of high throughput technologies open the door for researchers to evaluate the effects of multiple molecular features of gene on drug responses, identify reliable biomarkers and further build efficient predictors [4–6].

In the past decades, much effort has been devoted to the development of drug response prediction by means of genomic characterizations. Based on gene expression [7, 8], copy number variation (CNV) [9–12] and methylation [13, 14], a variety of approaches for screening biomarkers of drug response have been developed. For example, Zhang et al. [8] presented a method to identify significantly associated biomarkers and then developed ordinal genomic classifier using the hierarchical ordinal logistic model for predicting drug response. He et al. [11] provided a comprehensive review of the clinical relevance of CNVs to drug efficacy. There are also some existing data resources such as CancerDR [15], GEAR [16] and CARD [17] covering molecular signatures responsible for drug response. Despite the remarkable contribution to preclinical research, most of methods identifying biomarkers and predicting drug response with assumptions that genes act independently mainly, focused on single or multiple molecular alterations of patients while ignored functional relationships among genes within biological pathways. Drug response is not decided by several independent genes. It has been broadly accepted that alterations in signaling pathways largely determine the efficacy of kinase inhibitors used in the clinic [18], and in fact, the significance of pathways on drug efficacy has been recognized in recent pharmaceutical research [19]. Ammad-Ud-Din et al. [20] predicted drug response by inferring pathway-response associations with kernelized Bayesian matrix factorization. Wang et al. [21] constructed pathway-based models with four approaches inferring pathway activity derived from gene expression to predict drug response of cancer cells. Whereas the entire pathway is often too large to accurately interpret relevant pathological phenomena, a pivotal subpathway region representative of the corresponding entire pathway may be more effective and sensitive for dissecting the related phenomena [22, 23]. Furthermore, these existing methods mainly focused on only single omics data. Genome-wide multi-omics profiling of human cancers provides comprehensive information to identify biomarkers for improving the prediction of drug responses and to deepen our understanding of molecular mechanisms underlying drug actions. A few large scale cancer genome projects not only provide diverse molecular data but also drug response information of cancer patients such as The Cancer Genome Atlas (TCGA) (https://gdc-portal.nci.nih.gov/) and cancer cell lines [24], which provide new opportunities to identify signatures for individualized drug response prediction.

In this study, we aimed to identify reliable subpathway signatures for predicting anticancer drug response in cancer patients by simultaneously considering genetic and epigenetic (gene expression, CNV and DNA methylation) changes on the molecular states of pathway. By applying our method to five datasets, 46 subpathway signatures were identified to be associated with the responses to four drugs in different cancer types, the reliability of which has been demonstrated. Molecular characterizations of these subpathway signatures revealed essential roles of three omics types and the functional associations underlying different anticancer drug responses. Survival analysis suggested the clinical relevance of these subpathway signatures. Then, we applied the method to 191 anticancer drugs from CellMiner and uncovered their mechanism similarity based on subpathways we identified. Finally, the resource called CancerDAP (http://bio-bigdata.hrbmu.edu.cn/CancerDAP/) storing associations between these 191 anticancer drugs and 2751 subpathways at multi-omics levels, provides a flexible platform to explore molecular mechanisms of these anticancer drug responses from subpathway perspectives.

## Materials and methods

### Anticancer drug response datasets from TCGA

We collected the records of drug treatment in each cancer type from TCGA clinical data and manually standardized the drug names according to NCI drug dictionary and DrugBank [25]. In total, 46 candidate cancer-drug response datasets after the initial screening process were obtained, where the drug was antineoplastic agent based on DrugBank with usage frequency more than 50 individuals in corresponding cancer type. According to the RECIST standard [26], we classified the patients as responder (with complete response and partial response) and non-responder (with stable disease and progressive disease) for the specific drug (Additional file 1: Table S1). Given that imbalanced data could significantly compromise the performance of most standard learning algorithms [27], five cancer-drug response datasets were left, bladder urothelial carcinoma (BLCA)-cisplatin, BLCA-gemcitabine, Brain Lower Grade Glioma (LGG)-temozolomide, pancreatic adenocarcinoma (PAAD)-gemcitabine, stomach adenocarcinoma (STAD)-fluorouracil after excluding serious imbalanced datasets.

### Multi-omics data from TCGA

We collected three types molecular profiles, gene expression, CNV and DNA methylation, denoted as diverse molecular features of a gene from TCGA data portal (https://tcga-data.nci.nih.gov/tcga) for 4 cancer types (BLCA, LGG, PAAD, STAD). All molecular profiles for five datasets were level 3 data from TCGA. For each cancer type, we removed the sample without any one of three molecular features data or drug response record. Based on the fact that a single patient in TCGA may have been genomically profiled more than once, we calculated mean value of each gene in all for the patient in this condition. For expression, genes whose expression values were zero in more than 20% samples for each cancer type were removed. CNV profiles were the “all_data_by_genes.txt” tables from GISTIC 2.0 [28] applying to the masked copy number segment with default parameters. As regards DNA methylation from the Illumina Human Methylation 450 platform, we first excluded the CpG sites whose β-values had ‘NA’ greater than 20% in each cancer and filled using ‘impute’ package [29] for the remaining. Then, we calculated mean value and obtained a single value per gene if multiple CpG sites mapped to the same gene [30].

### Molecular profiles collection and preprocessing from CellMiner

The molecular profiles (gene expression, CNV and DNA methylation) of NCI-60 cell lines were downloaded from CellMiner [24] and preprocessing was similar to the above. In the project, cell line drug sensitivity was measured as negative log10 of the concentration at which the drug inhibited 50% of the cellular growth (processed GI50), and higher processed GI50 value indicates a better sensitivity of the cell line to a given drug. In total, 191 anticancer drugs were left for analysis after discarding the drugs where processed GI50 were ‘NA’ in more than 20% cell lines. For each anticancer drug, we divided cell lines into two groups (responder group with top 25% processed GI50 and non-responder with bottom 25% processed GI50) except the cell lines with ‘NA’ as processed GI50.

### Cancer hallmark dataset

We downloaded cancer hallmark gene sets from Gene Ontology (GO) Consortium [31] according to Plaisier et al. [32]. There are 35 GO sets that could be categorized into 10 cancer hallmarks. The GO data were downloaded from MSigDB database (http://software.broadinstitute.org/gsea/msigdb).

### Identifying subpathway signatures for anticancer drug response based on muti-omics data

**Fig. 1:**
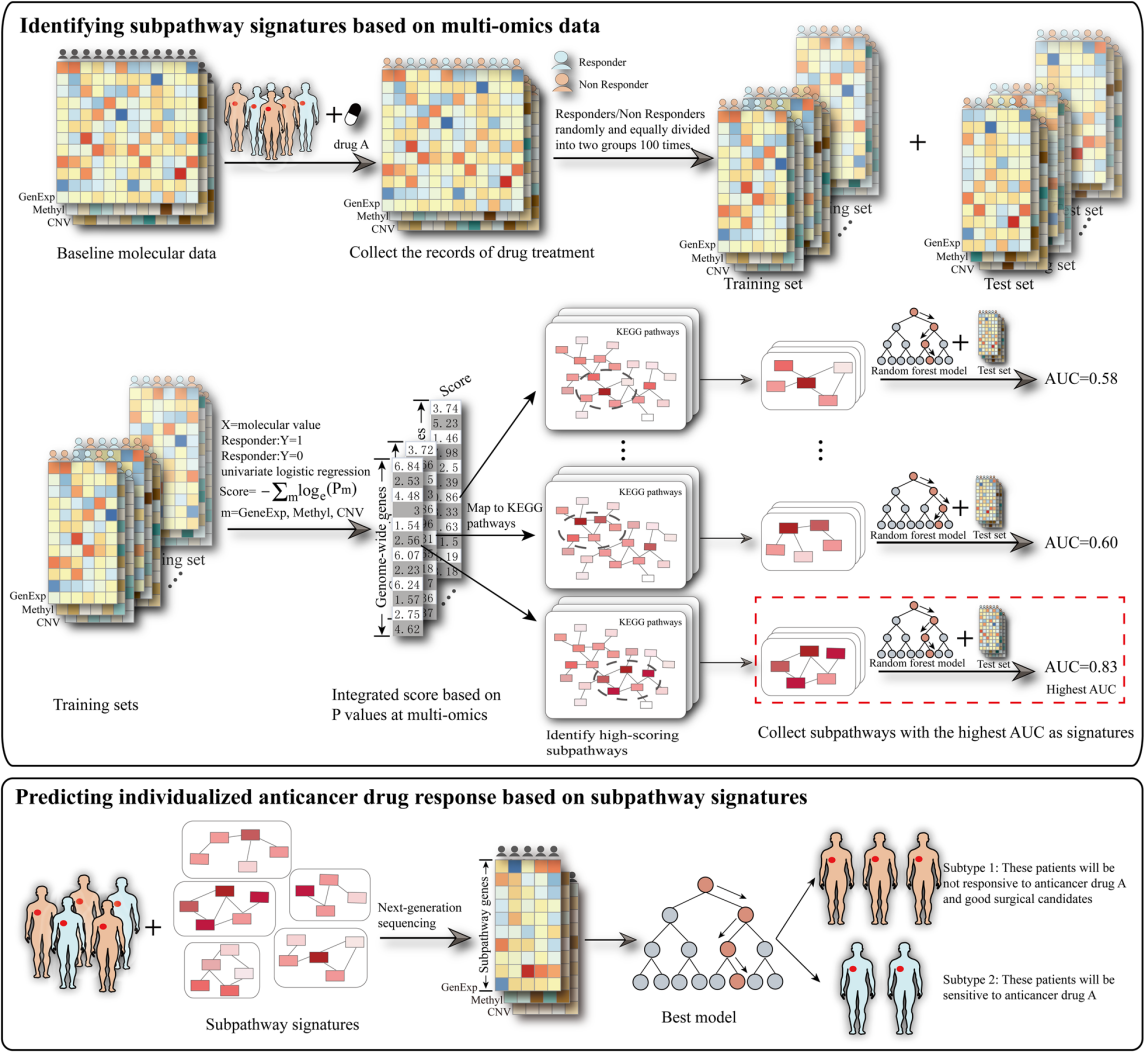
Schematic overview of the method to identify subpathway signatures for individualized anticancer drug response prediction by integrating multi-omics data

We first integrated multi-omic data and drug response records from TCGA. For a given drug-cancer response dataset, we distinguished the responders and non-responders into two groups equally at random, one for training set and the other for test set. The process of randomly grouping samples was repeated 100 times. For each sample grouping repeat, we first assessed the correlation of each gene with drug response at multi-omics level based on the corresponding training set. Next, we extracted subpathways associated with anticancer drug response by considering correlation between gene and anticancer drug response at multi-omics levels and pathway topologies. Then, we used multi-omics features of these subpathways as input into random forest model to predict drug response. The selected model was used to predict drug response in the corresponding test set and the area under the curve (AUC) of receiver operating characteristic (ROC) was calculated. Finally, subpathways used in the repeat with the highest AUC value were identified as subpathway signatures and the corresponding model was selected for predicting individualized response of the anticancer drug. The schematic overview of pipeline to identify subpathway signatures for predicting anticancer drug responses was shown in Fig. 1. For each grouping samples repeat of a given cancer-drug response dataset, the detailed processes are as follows.

#### Evaluating the correlation score between gene and drug response based on multi-omics data

We measured the correlation between each gene and response to the given drug by taking into account all three molecular features (mRNA expression, CNV, DNA methylation). We first fitted the univariate logistic regression model to evaluate the correlation between drug response and the gene at different omics levels respectively. Then, we derived a combined score for each gene, which was summarized as the sum negative natural logarithm of single molecular feature P-values [33]. The formula is as follows:

$${\text{Score}} = - \sum_{\text{m}} \log_{\text{e}} \left( {{\text{P}}_{\text{m}} } \right), \;{\text{m}} = {\text{gene}}\;{\text{expression}},\; {\text{CNV}},\; {\text{methylation}}$$

*P*_*m*_ represents the P value from the univariate logistic regression based on gene expression, CNV and methylation respectively.

#### Locating subpathways most relevant to drug response

Next, the score list and KEGG [33] pathway data (preprocessing as we previously described [34]) were used as the input into signet [35], which searches for high-scoring subpathway regions of genes within pathways based on simulated annealing algorithm and test their significance. Only the subpathways whose P values from signet passed the cutoff of P < 0.05 and members contained at least three connected genes were identified as candidates to construct predictive model of drug response.

#### Inferring patient-specific subpathway activities at different omics levels

To weight each of these selected subpathways in training set, we introduced a measure to infer patient-specific subpathway activities at different omics levels. Activity of subpathways associated with drug response in patient p was assessed according to the function [36, 37].

$${\text{Z}}_{\text{sp}} = \left( {\frac{{\sum\nolimits_{\text{i}}^{\text{n}} {{\text{X}}_{\text{i}}\upbeta_{\text{i}} } }}{\text{n}} - \frac{{\sum\nolimits_{\text{i}}^{\text{N}} {{\text{X}}_{\text{i}}\upbeta_{\text{i}} } }}{\text{N}}} \right)\frac{{\sqrt {\left| {\text{n}} \right|} }}{{\upsigma_{\text{p}} }}$$

where n is the number of genes in the subpathway and N is the number of all genes detected in sample p. X_i_ represents the expression value (CNV and methylation value respectively) of the gene i in sample p. β_i_ represents the estimated regression coefficient of gene i in the univariate logistic regression model. σ_p_ is the standard deviation of all the genes values X_i_ multiplying β_i_ in sample p.

We therefore could acquire three activity matrixes from gene expression, CNV and DNA methylation of subpathways-associated drug response in training set.

#### Identification of the subpathway signatures

The integrated matrix that combined above three activity matrixes was used as features to construct random forest model for predicting drug response of cancer patients with the R package randomForest [38]. For each random forest, 10,000 decision trees were generated. This model was further employed to predict the drug response of patients in test set based on integrated matrix using the estimated regression coefficients of genes and selected subpathways from training set. Performance evaluation of prediction model was displayed using receiver operating characteristic (ROC) curve and assessed by the area under the curve (AUC) of ROC with the R package pROC [39]. Finally, subpathways used in the repeat with the highest AUC value in test set were identified as subpathway signatures and the corresponding model was selected for predicting individualized drug response of cancer patient.

### Construction of models to validate the prediction power of subpathway signatures

To evaluate whether combining multi-omics and subpathway information could improve the prediction power of drug sensitivity, we constructed two additional models, ‘Gene with pathway’ and ‘Gene without pathway’ models. Gene with pathway model was only based on gene expression. The gene score as input into signet was negative natural logarithm of P-values from the univariate logistic regression and subpathway activity matrix for random forest was from gene expression. Gene without pathway model was constructed just using genes whose P values were less than 0.05 and the value in activity matrix for random forest is expression value multiplying corresponding estimated regression coefficient from the univariate logistic regression.

### Survival analysis

For evaluating the associations of subpathways related to drug response with patients’ overall survival at three omics levels, we applied Cox proportional hazards model to subpathway activity from gene expression (CNV, DNA methylations respectively). When exploring the prognostic value of subpathways as a whole, we grouped patients using K-means algorithm based on combined subpathway activity matrixes (k = 2). Then, we used Kaplan–Meier survival curve and log-rank test to assess the survival difference.

## Results

### The performance of subpathway signatures for predicting individualized drug response

**Fig. 2:**
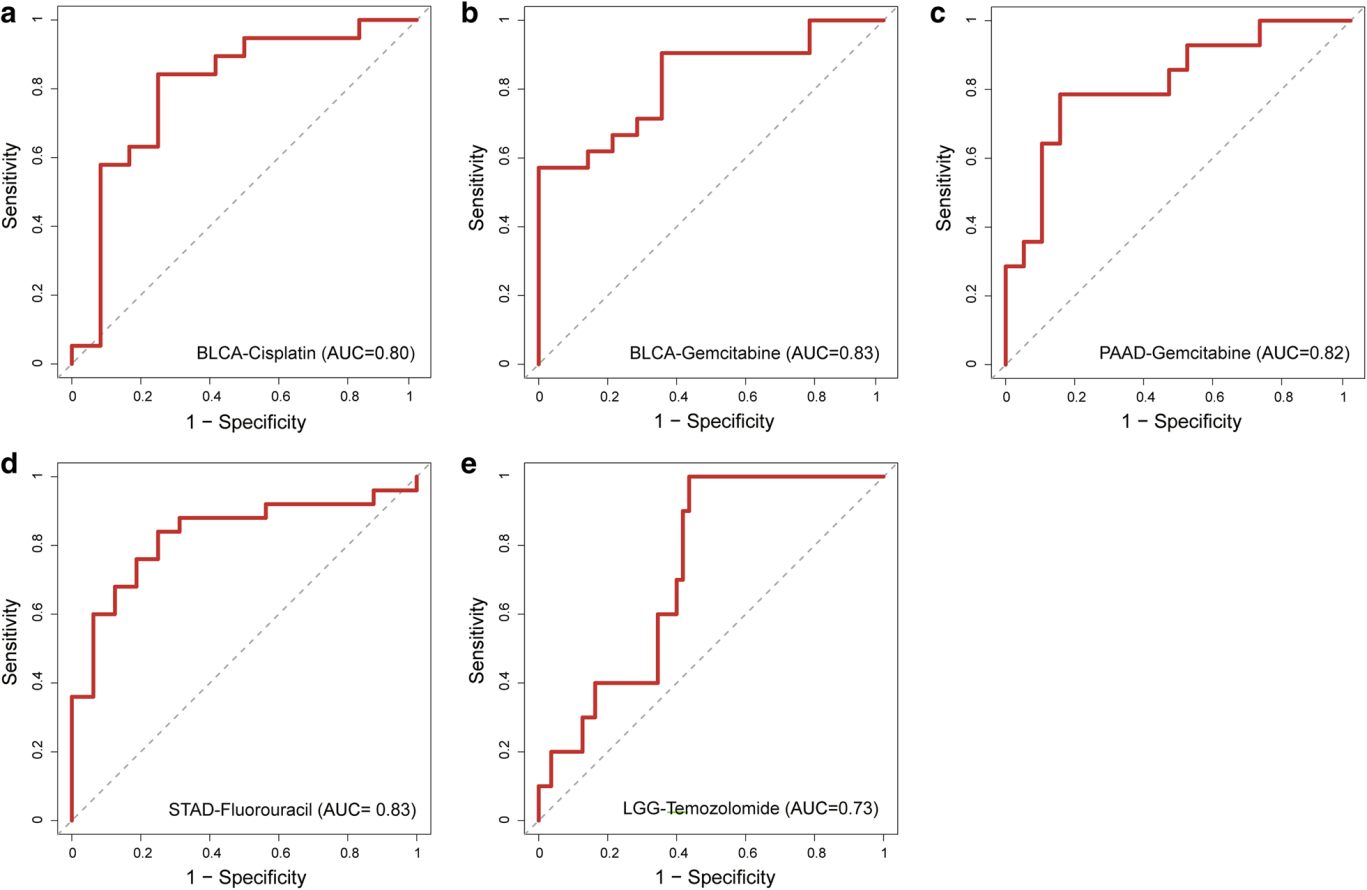
Predictive power of subpathway signatures across five cancer-drug response datasets from TCGA, including **a** BLCA-cisplatin, **b** BLCA-gemcitabine, **c** PAAD-gemcitabine, **d** STAD-fluorouracil and **e** LGG-temozolomide

We applied our method to five anticancer drug response datasets. In total, 46 subpathway signatures were identified for four anticancer drugs in different cancer types (Additional file 2: Table S2). We first examined the predictive performance of these subpathway signatures. Figure 2 shows the ROC curves of these subpathway signatures for predicting drug response of cancer patients in test sets of the five datasets using random forest model. We found that all five datasets show relative high predictive performances. The AUC values range from 0.73 to 0.83 and the highest AUC value is 0.83 in BLCA-gemcitabine and STAD-fluorouracil datasets. We next investigated how the member genes within subpathway signatures used to construct drug response prediction model differ between responder and non-responder groups in three molecular feature types (Additional file 3: Figure S1). As seen in the figure, there is nearly no difference between responder and non-responder groups in whichever molecule feature of three, implying that pathway data could provide additional information to infer the individual’s drug response. The result suggests that pathway topological structure embracing some hidden meaningful information could be utilized to explore associations between genomic data and drug response.

**Fig. 3:**
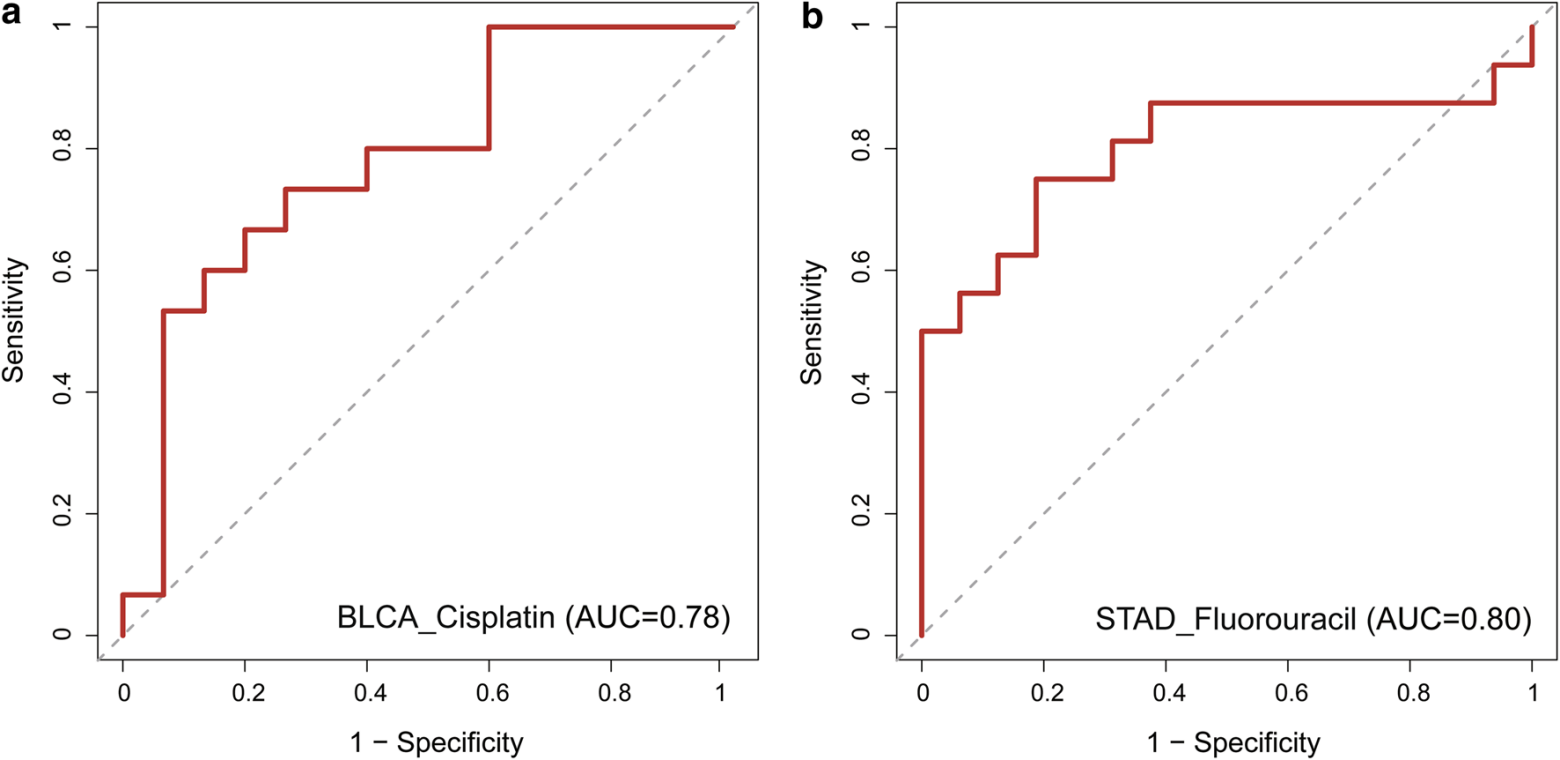
Validation of the predictive power of identified subpathway signatures in two independent datasets from CellMiner by training on datasets from TCGA, including **a** cisplatin and **b** fluorouracil

Then, we further validated the prediction power of these subpathway signatures in two independent datasets from CellMiner, considering the availability of data (seen in “Materials and methods”). We evaluated the predictive performances of subpathway signatures of two anticancer drugs including cisplatin and fluorouracil. For the cell line datasets, the top 15 cell lines with the largest GI50 values were assigned to the “responder” group and 15 cell lines with the lowest GI50 values were defined as “non-responder”. We used subpathway signatures and the corresponding random forest model selected in TCGA datasets of these two drugs to predict cellular responses based on the above independent datasets respectively. The results showed that our identified subpathway signatures also exhibited relative high predictive power in both cisplatin (AUC = 0.78) and fluorouracil (AUC = 0.80) datasets (Fig. 3). Taken together, these results demonstrate the reliability of these identified subpathway signatures for individualized anticancer drug response prediction.

### Comparisons of the predictive power of the multi-omics subpathway signatures with single omics and gene-based signatures

**Fig. 4:**
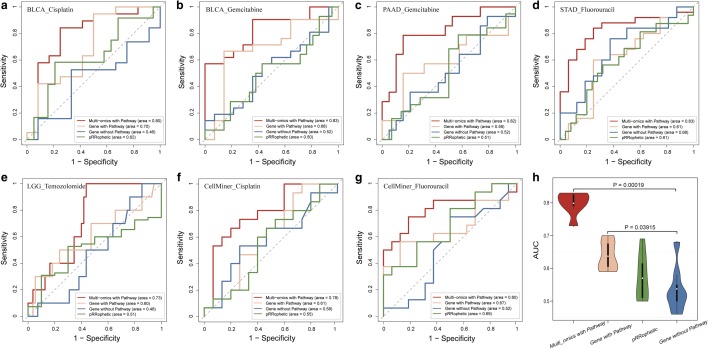
Performance comparisons of drug response prediction models based on the identified multi-omics subpathway signatures (multi-omics with pathway), single omics subpathway signatures (gene with pathway) and gene signatures (gene without pathway and pRRophetic), respectively. ROC curves in the **a** BLCA_cisplatin, **b** BLCA_gemcitabine, **c** PAAD_gemcitabine, **d** STAD_fluorouracil, **e** LGG_temozolomide, **f** CellMiner_cisplatin and **g** CellMiner_fluorouracil. **h** Violin plots indicate the AUCs distribution and average values (white dot) of each model in seven datasets. P-values are the maximum P-values of the AUCs of Multi-omics with Pathway versus the AUCs of remaining three, and the AUCs of Gene with Pathway versus the AUCs of remaining two by the paired t-test

Since many existing drug response prediction approaches based on gene expression, to investigate whether the identified signatures based on our method combining multi-omics data and subpathway information adds predictive power, we challenged our multi-omic subpathway signatures for drug response prediction against other three models: (1) gene without pathway model constructed based on only gene expression data (details in “Materials and methods”), (2) gene with pathway model constructed from gene expression data and pathway information (details in “Materials and methods”) and (3) pRRophetic [40], which was an existing method for predicting clinical chemotherapeutic response based on tumor gene expression data. As a result, all these three models show limited performances compared with our multi-omics subpathway signatures based model in seven datasets (Fig. 4). The subpathway signature-based model significantly outperformed Gene with Pathway model which incorporated pathway topological structure but focused on single omics, pRRophetic and Gene without Pathway model which ignored pathway information with maximum P value = 0.00019 among the paired t-test (Fig. 4h). In addition, the Gene with Pathway is significantly better than Gene without Pathway (paired t-test: P-value = 0.039) and pRRophetic [40] method (paired t-test: P-value = 0.035) (Fig. 4h). Obviously, combining multi-omic data and pathway topological structure information could improve the performance on predicting anticancer drug responses. These above results confirm the superiority of our subpathway signatures and the significance of integrating multi-omics and subpathway information for predicting drug response of cancer patients.

### Dissecting subpathway signatures related to anticancer drug response

**Fig. 5:**
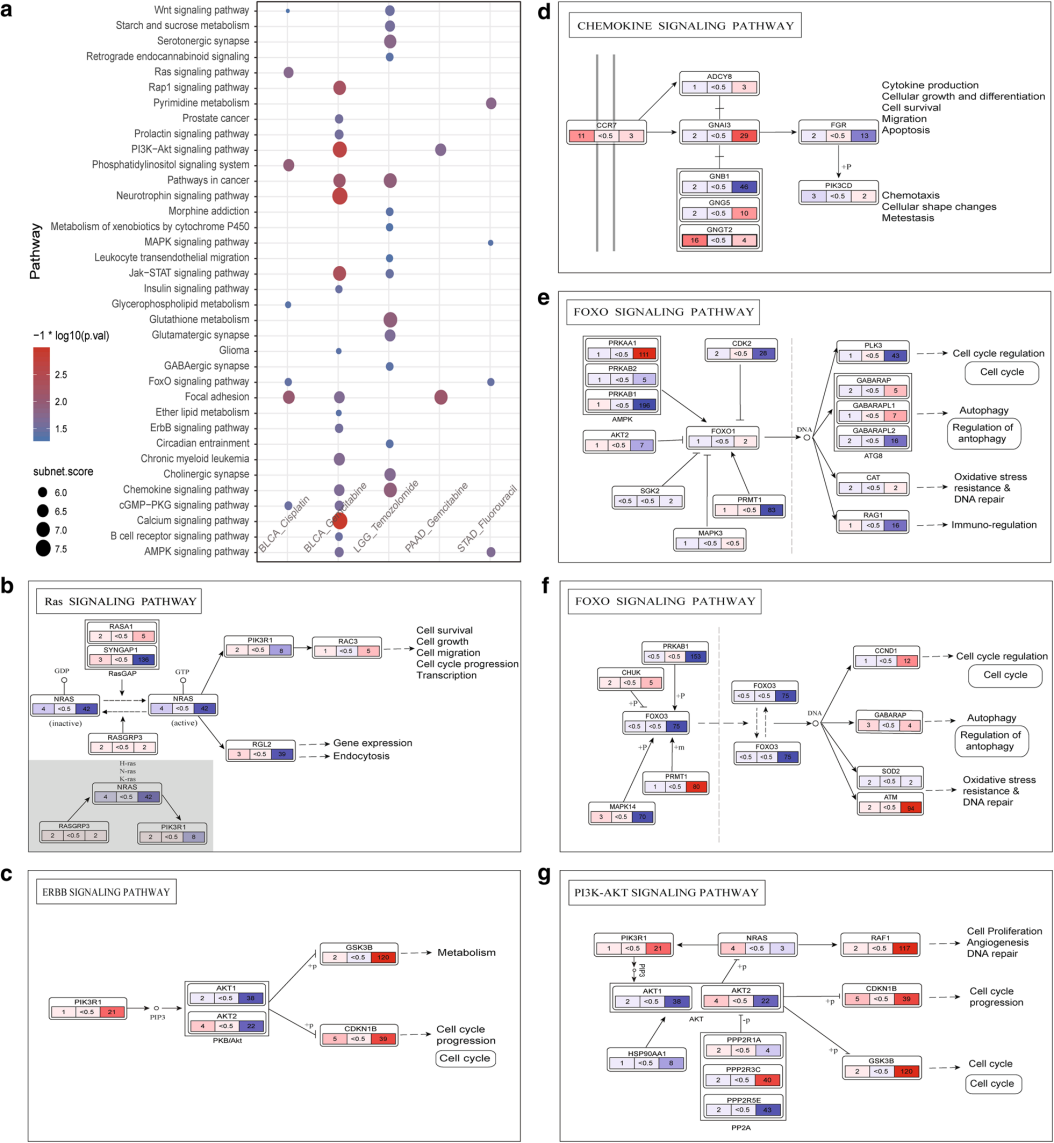
Molecular characterization of subpathway signatures. **a** Dot plot of scores and P values from signet for each of 46 subpathway signatures associated with the responses of different drugs, with y-axis representing entire pathways in KEGG where subpathways are located in. Each node represents the subpathway derived from entire pathway in corresponding dataset. The node size indicates the score of subpathway. Color intensity of node corresponds to negative natural logarithm of P-value, and redder represents the more statistically significant. **b**–**g** Structure visualization of several subpathway signatures. The rectangle symbolizing gene is divided into three parts, representing left to right: CNV, gene expression, methylation. The number and color intensity of subrectangle correspond to correlation degree of gene with drug response estimated by univariate logistic regression model. Red depicts positive and blue depicts negative correlation

We then explored the correlation between subpathways and anticancer drug response at molecular level. In total, 46 subpathway signatures associated with anticancer drug response, were identified from five datasets, BLCA-cisplatin, BLCA-gemcitabine, LGG-temozolomide, PAAD-gemcitabine, STAD-fluorouracil. Several entire pathways in which these subpathways are located, are well-known to mediate antidrug response, such as Wnt/β-catenin signaling pathway [41, 42], Ras signaling pathway [43–45], PI3K-Akt signaling pathway [46, 47], MAPK signaling pathway [48], Jak-STAT signaling pathway [49] and AMPK signaling pathway [50]. Figure 5a provides the summary of these 46 subpathway signatures. Calcium signaling pathway which has been reported to play essential roles in acquired multidrug resistance of cancer cells [51] is the first top-scoring subpathway location, followed by neurotrophin signaling pathway (Fig. 5a). Overall, subpathway signatures are associated with diverse drug response differed greatly. Focal adhesion is most commonly found only in BLCA-cisplatin, BLCA-gemcitabine, PAAD-gemcitabine. In addition, while some anticancer drugs are widely used in multiple cancers just like gemcitabine in BLCA and PAAD, there are considerable differences in between 18 and 2 subpathway signatures in BLCA-gemcitabine and PAAD-gemcitabine respectively. This suggested that the mechanism of drug response may differ across diverse cancer types due to variable levels of genomic instability and heterogeneity.

To further characterize how these subpathways could contribute to drug response at multiple molecular layers (gene expression, CNV and methylation), the topological structures of some pathways were extracted (Fig. 5b–g). Subpathway signatures covering important genes, interaction partners and regulation patterns could provide potential insights into mechanisms underlying drug response. These subpathways reflect the diverse cellular events associated with anticancer drug response, such as cell survival, cell growth, cell cycle, apoptosis, autophagy, metabolism, immuno-regulation, DNA repair (Fig. 5b–g), consistent with our prior knowledge. More details, we dissected the correlation between member genes within subpathway and drug response at multi-omics levels. In each subpathway, the correlations between the same member gene and drug response are different or even opposite on three molecular features, gene expression, CNV and methylation. For example, RasGAP in subpathway from Ras signaling pathway, PKB/Akt in subpathway from ERBB signaling pathway, AKT in subpathway from PI3K-AKT signaling pathway, AMPK in subpathway from FOXO signaling pathway. These findings indicate that both genetic and epigenetic events play considerable roles in anticancer drug response and combining data from different omics may provide more accurate and comprehensive information to predict drug response of individuals.

In addition, we constructed a subpathway functional similarity network to systematically understand the linkage of drug response mechanisms. We inferred functional similarity between two subpathways in virtue of semantic similarity acquired from R package GOSemSim [52]. Two subpathways were connected if the semantic similarity of subpathway–subpathway reached 0.6 (Additional file 4: Figure S2A). There are strong connections between subpathways associated with different anticancer drug responses, especially the subpathway derived from MAPK signaling pathway in STAD-fluorouracil (inter: 97%, intra: 3%) (Additional file 4: Figure S2B). This suggests that despite relatively small intersection in subpatwhay locations (Fig. 5a), there are similar cellular functions underlying different drug responses, providing opportunity for developing drug repositioning. We also dissected relations between subpathways associated with drug sensitivity and hallmarks of cancer. Most subpathways related to at least one hallmark are identified (32/46), suggesting that some subpathways influence on drug sensitivity as well as mediating the development of cancer.

### Prognostic potential of the subpathway signatures involved in drug response

**Fig. 6:**
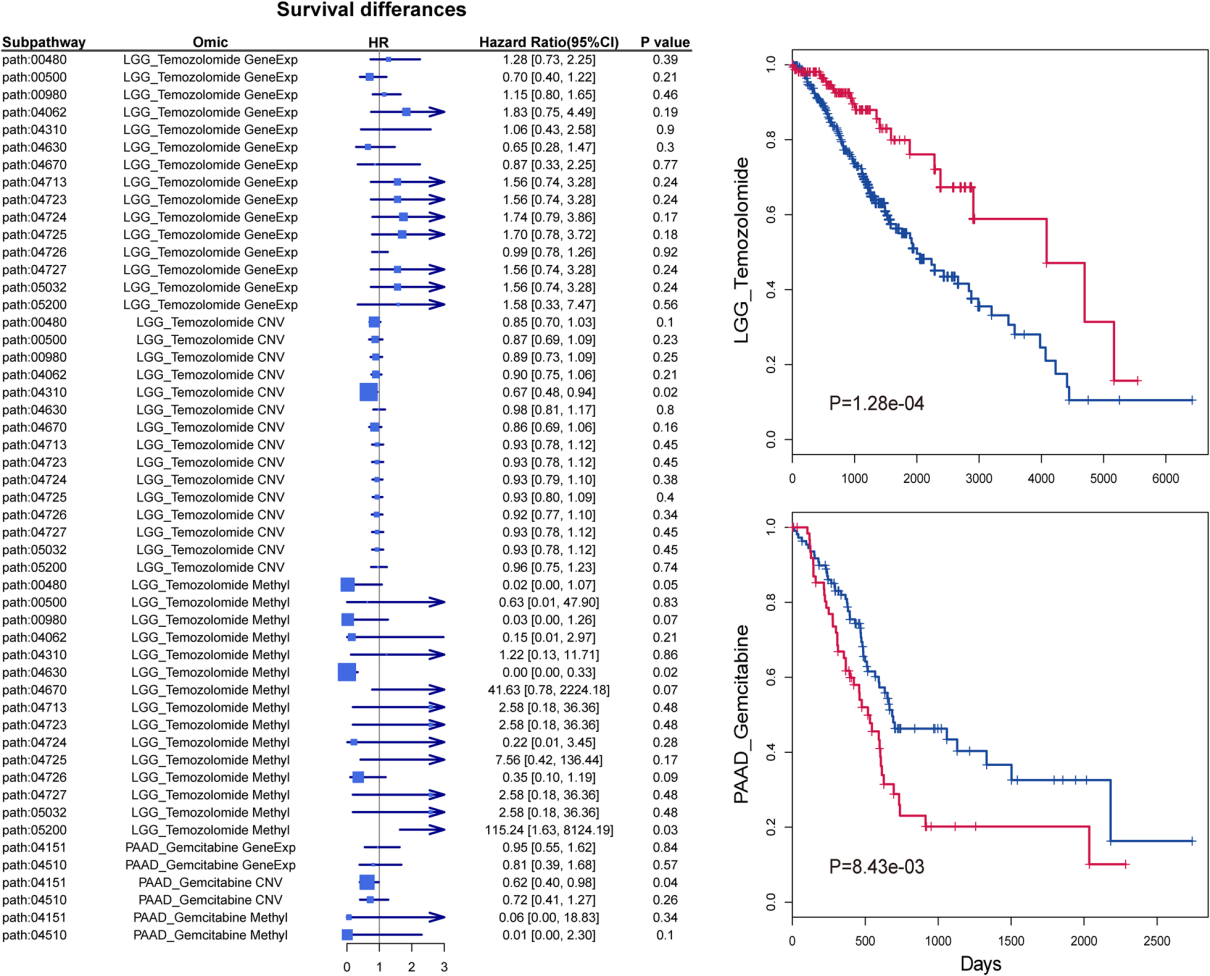
Discriminative prognosis power of the identified subpathway signatures. Left: Forest plot shows HR and 95% CI from univariate Cox proportional hazards model of the subpathway signatures at three omic level from LGG-temozolomide and PAAD-gemcitabine. Right: Kaplan–Meier survival plots of patients grouped by K-means based on combined activities with multi-omics of subpathway signatures in LGG and PAAD. The P values were estimated based on log rank test

We examined whether these subpathway signatures could help to stratify patients into distinct clusters or subtypes that were linked to survival. We first collected four datasets consisting of the same three omics data consistent with previous of patients with their survival information from TCGA, including BLCA, LGG, PAAD, STAD, and followed previous steps to infer activity of subpathway signatures identified in corresponding drug sensitivity analysis. Then, we fit cox proportional hazards regression model for each subpathway based on inferred activity. The hazard ratio (HR) is a measure of the relative survival relevance for each molecular feature of subpathway, where an HR > 1 represents a risk factor and indicates that the higher level activity of subpathway is associated with shorter survival. There are 36 subpathways (including the same subpathway at different molecular features) with a hazard ratio (HR) > 1 and 102 with a HR < 1, 19 significantly associated with survival outcome (P < 0.05) (Fig. 6a and Additional file 5: Figure S3), such as subpathways involved in Wnt signaling pathway on expression level, Ras signaling pathway on methylation level, ErbB signaling pathway on copy number variation level from BLCA-cisplatin. Then, to investigate if combined activities with multi-omics of subpathway could contribute to predict clinical outcome, we performed Kaplan–Meier survival analysis based on the groups divided by K-means using the multi-omics activities matrix of corresponding subpathways. It’s worth to note that combining all subpathway signatures could be used as prognostic biomarkers for better patient stratification with increased significance in LGG (P = 1.28 × 10^−4^) and PAAD (P = 8.43 × 10^−3^) (Fig. 6b). These results indicate that potential uses of subpathway signatures involved in anticancer drug response as prognostic markers and provide novel insights for future development of survival prediction and patient stratification.

### A landscape of subpathway for 191 anticancer drug responses in cancer cell lines

**Fig. 7:**
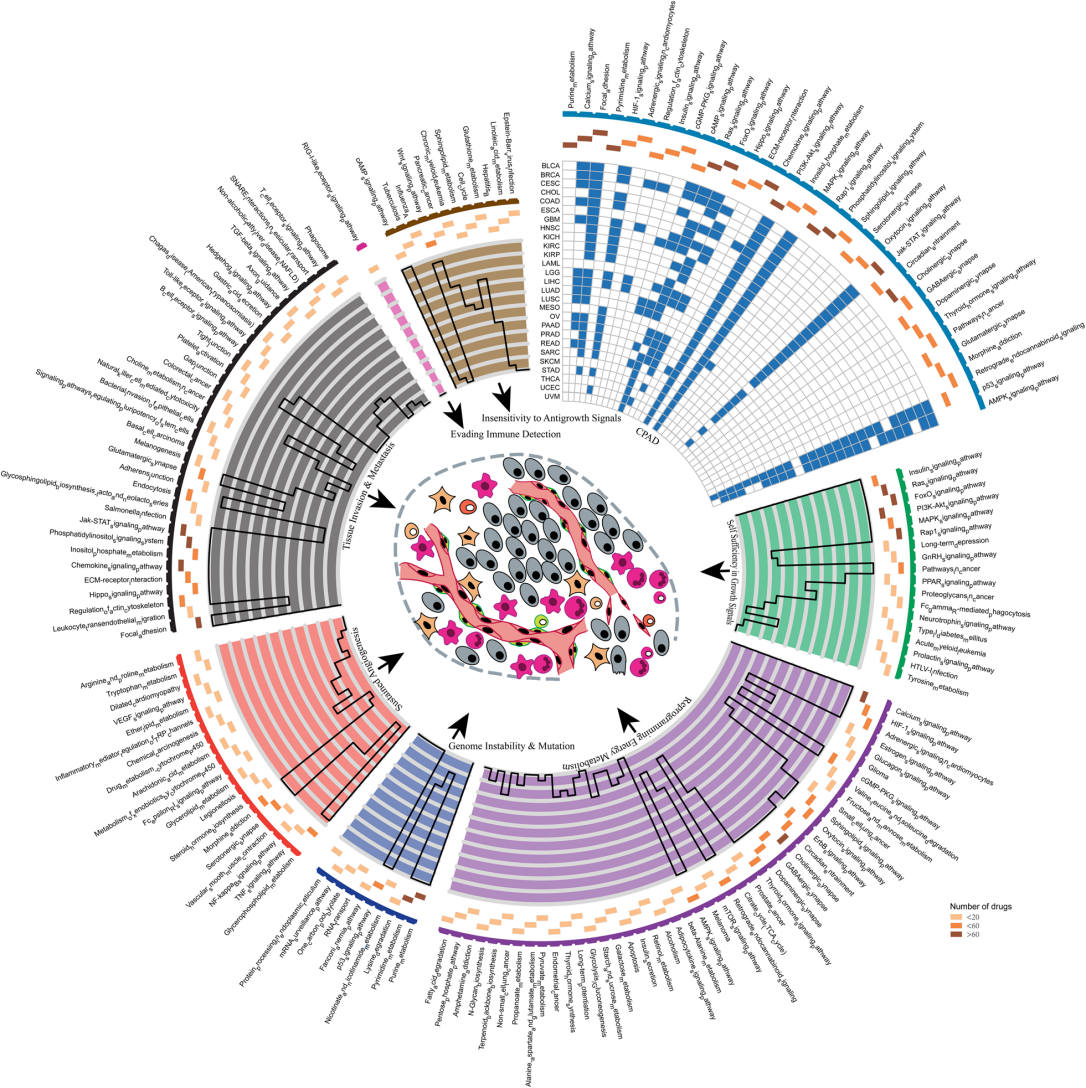
The landscape of subpathways associated with the response of 191 anticancer drugs in CellMiner. Overview of the 141 entire pathways embracing all subpathways related with the responses of 191 anticancer drugs. Each sector indicates a hallmark of cancer with pathways related to that hallmark listed. The top right sector shows the associations between top 25% pathways ranking by the number of anticancer drugs affected and TCGA cancer types. Blue compartment indicates that the association between pathway and cancer has been verified by experiments. The next ring and histogram (where the y axis limit is 23) illustrate the number of anticancer drug affected

The availability of public pharmacogenomic resources with drug response information has made it possible to large scale screen subpathways associated with drug response and further guide the early phase clinical trials of anticancer drugs development. CellMiner is such a database that makes available multiple genomic and pharmacological data sets for the NCI-60 cell lines. Then, we identified subpathways associated with the responses to anticancer drugs from CellMiner based on multidimensional molecular profiles (gene expression, CNV, methylation) and drug activities data of 60 diverse human cancer cell lines. In all, 2751 subpathways from 141 entire KEGG pathways were identified to be associated with 191 anticancer drug responses. Then, a landscape of subpathways related to anticancer drug responses was constructed (Fig. 7). Most entire pathways (116 out of 141 pathways) are related with responses of more than one anticancer drug. There are five pathways associated with more than 100 anticancer drug responses, including Jak-STAT signaling pathway (111), FoxO signaling pathway (112), pyrimidine metabolism (114), focal adhesion (138) and purine metabolism (176). In contrast, some pathways are specifically related with anticancer drug response, such as amphetamine addiction in cabozantinib, and mRNA surveillance pathway in gefitinib. Next, we dissected associations between these subpathway and the development of cancer. The semantic similarity between pathways and cancer hallmarks were calculated. We found that most of these pathways from which subpathway originated, were functionally related with cancer hallmarks including ‘reprogramming energy metabolism’, ‘tissue invasion and metastasis’ and ‘sustained angiogenesis’ (Fig. 7). Furthermore, we focused on the roles of pathways related to more than 25% anticancer drug responses in tumorigenesis and found that some of them were experimentally validated to be associated with many cancer types according to a reliable database CPAD [53]. Especially, some notable oncogenic pathways are consistently implicated in most cancers, for example, AMPK signaling pathway, p53 signaling pathway, Jak-STAT signaling pathway and MAPK signaling pathway. This is consistent with the above mentioned result that some pathways affect drug response as well as mediate tumorigenesis and cancer progression. Their oncogenic roles have been well-reported but much less known about their effects in drug response, and these pathways involved in cancers are worthy to be further investigated in drug response.

Evaluation of clinical drug–drug similarity has many potential applications in various fields, such as mechanism of drug action, drug response prediction and drug repositioning [54]. Here, we assessed drug–drug similarity based on subpathway identified for these 191 anticancer drug responses. First, we constructed a similarity matrix by calculating the mean semantic similarity score between subpathways for drug A and subpathways for drug B as similarity score between two drugs, and then performed hierarchical clustering (Additional file 6: Figure S4). We found that some anticancer drugs with identical mechanism of action recorded in CellMiner tended to be clustered together (arrow in Additional file 6: Figure S4). For example, most anticancer drugs whose mechanisms of action are topoisomerase 2 inhibitor are assigned to the same cluster. This finding suggests the subpathway could unclose drug similarity, facilitate the mechanism study of drug response and further provide guidance for individualized treatment of cancer.

### CancerDAP: an online database molecularly characterizing subpathways involved in cellular response to 191 anticancer drugs

To further promote investigating mechanism of drug response, we constructed a convenient and friendly database called CancerDAP (The Anticancer Drug Active subPathway database), which provides detailed characterization of the effects of subpathways and member genes in anticancer drug response from a multi-dimensional perspective. The database stores 2751 subpathways associated with 191 anticancer drug responses. All data in CancerDAP were organized using MySQL. The CancerDAP database is available at http://bio-bigdata.hrbmu.edu.cn/CancerDAP/.

**Fig. 8:**
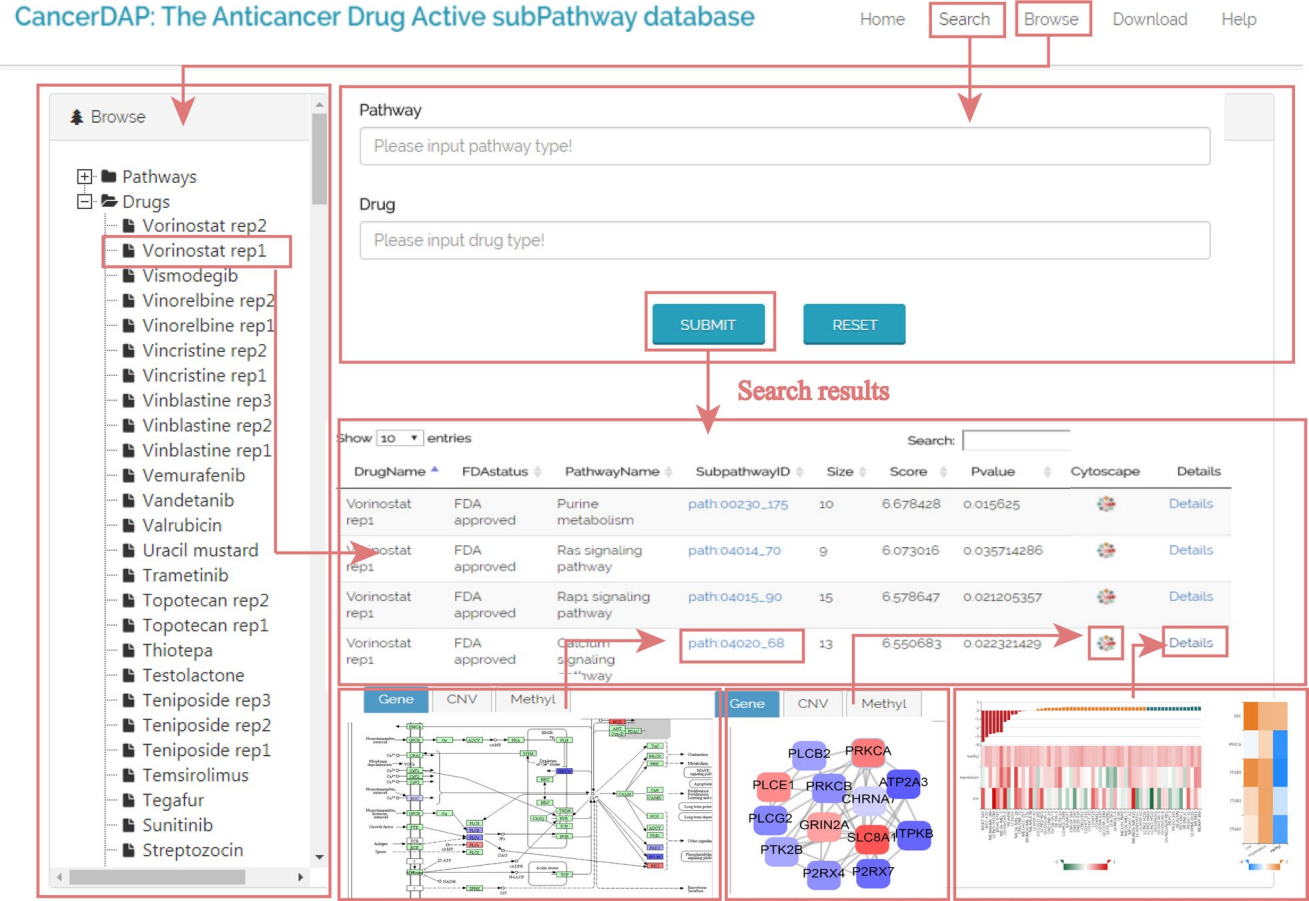
A schematic workflow of CancerDAP

CancerDAP provides a user-friendly interface mainly consisting of three modules: Search, Browse and Download (Fig. 8). The “Search” module allows users to search by pathways of interest, anticancer drugs of interest, or both. In the “Browse” module, when selecting a specific pathway or drug, query result presents basic annotations for each entry, including drug name, FDA status and attributes of subpathway (entire pathway name, subpathwayID, size, score and P value). Besides, two hyperlinks to KEGG and Cytoscape to get subpathway structure visualizations. Furthermore, there is a hyperlink named “details” in this module providing data visualization for multi-omic subpathway activity and the correlation between member genes and corresponding anticancer drug response across NCI-60 cell lines. Intuitive images in CancerDAP can offer insights into the effects of subpathway and member genes on anticancer drug sensitivity at molecular level. The “Download” module allows users to freely obtain the comprehensive data of all subpathways for analysis. Besides, there is also a “Help” module. For the first-time user, the module provides a detailed guide to search and browse through the resource to retrieve the desired information.

## Discussion

Identifying biomarkers for clinical anticancer drug response prediction based on molecular data is an essential step for personalized medicine [55–58]. Although a number of methods for identifying signatures and predicting drug response were proposed, many of them have been limited to overlook interaction of genes in biological pathway or only focus on one aspect of gene alterations [4, 6, 59–62]. Here, we proposed a computational method to identify subpathway signatures for predicting anticancer drug response based on multi-omic data. Our method not only identified subpathway signatures which incorporate functional interactions between genes but also simultaneously considered the multi-omic effects (gene expression, CNV and methylation) of genes on drug responses.

The method was applied to identify subpathway signatures associated with the responses of four anticancer drugs (cisplatin, gemcitabine, temozolomide and fluorouracil) in different cancer types. In total, 46 subpathway signatures were identified and the predictive powers of these signatures for individual drug responses were validated. We first compared the predictive power of these subpathway signatures with gene signatures, which is one of the most commonly used strategies for drug response prediction. Subpathway signatures exhibited better predictive performance across seven drug response datasets used in this study. Then, we also validated the necessity of integrating multi-omics data to identify drug response signature. All these results highlight the superiority and reliability of drug response related subpathway signatures we identified, suggesting that incorporating multi-omic data with pathway topological structure embracing some hidden meaningful information could improve the performance of individualized drug response prediction. These reliable subpathway signatures reported here have profound implications for personalized medicine and drug development, where our approach may have the potential to be used for evaluating the patient’s response to medications prior to carrying out clinical trials.

Analysis of 46 subpathway signatures provides novel insights into the underlying mechanisms of anticancer drug response. We found that three molecule features (expression, CNV, methhylation) of genes exhibited essential but inconsistent effect modes on drug response. This finding reveals that different molecular aberrations can give rise to a single clinical phenotype, proving the necessity and validity of integrating multi-omic data [63, 64]. Moreover, strong functional similarity between subpathway signatures from different drug response datasets despite locating to different entire pathways, indicates that subpathways may play similar roles across tumor types under different medication. Most subpathways are related to at least one hallmark, suggesting that some subpathways influence on drug sensitivity as well as mediating the development of cancer. Some of 46 subpathways showed prognostic power, and the subpathways from LGG-temozolomide and PAAD-gemcitabine as a whole could discriminate patients with significantly different outcomes.

Large scale screening the subpatwhays associated with anticancer drug sensitivity provides global insights and facilitates to unclose link among drug response mechanisms. We also provided an important resource of 2751 subpatwhays related to all 191 anticancer drugs documented in CellMiner. Drug similarity analysis based on these subpathways efficiently identified the anticancer drugs with similar mode of action and hence exhibited many potential applications in drug development. To further facilitate the translational application of study, the comprehensive information about 191 anticancer drugs and their response related 2751 subpathways have been compiled, curated, and presented in a freely accessible resource named CancerDAP (http://bio-bigdata.hrbmu.edu.cn/CancerDAP/).

## Conclusions

In summary, our study identified and dissected subpathway signatures for individualized anticancer drug response, which provides useful resources to promote the precision cancer therapy and molecular mechanism study for drug responses.

## Additional files

**Additional file 1: Table S1.** The number of responders and non responders in TCGA datasets.

**Additional file 2: Table S2.** The subpathway signatures associated with four anticancer drugs response.

**Additional file 3: Figure S1.** Multi-omics characterization of member genes involved in the 46 subpathway signatures. The three molecular omics of genes, which always appear in the same order, are, from top to bottom: gene expression (GeneExp), CNV and methylation. The entries in heatmap are expression (CNV or methylation) levels of genes from TCGA.

**Additional file 4: Figure S2.** Functional connections between subpathway signatures associated with the responses of different drugs. (A) Functional similarity network of subpathway signatures identified for different anticancer drugs in various tumor types. Node indicates subpathway and edge indicates the semantic similarity score greater than 0.6 from GOSemSim between two subpatwhays. Pie chart in the node indicates pathway related cancer hallmarks. (B) The degree distribution of nodes in (A). Dark blue represents the number of intra connections between subpathway signatures from the same dataset. Light blue indicates the number of inter connections between subpathway signatures from different datasets.

**Additional file 5: Figure S3.** Discriminative prognosis power of the identified subpathway signatures. (A) Forest plot indicates HRs and 95% CI from univariate Cox proportional hazards model of the subpathway signatures at three omic level from BLCA-Cisplatin, BLCA-Gemcitabine and STAD-Fluorouracil.

**Additional file 6: Figure S4.** Unsupervised hierarchical clustering of drugs based on the mean semantic similarity scores of subpathways related with their responses.

## Data Availability

The multi-omics profiles and anticancer drug response data of these four cancer types are available at TCGA data portal (https://tcga-data.nci.nih.gov/tcga). The multi-omics profiles and drug sensitivity data of cell lines are available at CellMiner database (https://discover.nci.nih.gov/cellminer/).

## Abbreviations

CNV: copy number variation
TCGA: The Cancer Genome Atlas
BLCA: bladder urothelial carcinoma
LGG: Brain Lower Grade Glioma
PAAD: pancreatic adenocarcinoma
STAD: stomach adenocarcinoma
AUC: area under the curve
ROC: receiver operating characteristic
HR: hazard ratio
CI: confidence intervals
