# Supplementary material for: Identifying subpathway signatures for individualized anticancer drug response by integrating multi-omics data
Source: J Transl Med. 2019 Aug 6;17:255. doi: 10.1186/s12967-019-2010-4 (PMC6685260; doi:10.1186/s12967-019-2010-4)
Supplement: Supplementary file 1 — Additional file 1: Table S1. The number of responders and non responders in TCGA datasets. [file 12967_2019_2010_MOESM1_ESM.docx]

**Table S1.** The number of responders and non responders in TCGA

| CancerType | DrugName | Responder | Non-responder |
| --- | --- | --- | --- |
| BLCA | Gemcitabine | 42 | 29 |
| BLCA | Cisplatin | 39 | 24 |
| BRCA | Cyclophosphamide | 113 | 8 |
| BRCA | Doxorubicin | 70 | 7 |
| BRCA | Paclitaxel | 46 | 10 |
| BRCA | Docetaxel | 47 | 6 |
| CESC | Cisplatin | 55 | 9 |
| HNSC | Cisplatin | 45 | 5 |
| LGG | Temozolomide | 20 | 110 |
| PAAD | Gemcitabine | 29 | 38 |
| STAD | Fluorouracil | 51 | 32 |
| TGCT | Etoposide | 51 | 1 |
| TGCT | Bleomycin | 49 | 2 |
| TGCT | Cisplatin | 50 | 0 |

The number is intersection of patients with drug response records and patients with gene expression, CNV and methylation.
