# Supplementary material for: Identifying subpathway signatures for individualized anticancer drug response by integrating multi-omics data
Source: J Transl Med. 2019 Aug 6;17:255. doi: 10.1186/s12967-019-2010-4 (PMC6685260; doi:10.1186/s12967-019-2010-4)
Supplement: Supplementary file 2 — Additional file 2: Table S2. The subpathway signatures associated with four anticancer drugs response. [file 12967_2019_2010_MOESM2_ESM.docx]

**Table S2.** The subpathway signatures associated with four anticancer drugs response

| Subpathway | Size | Entire PathwayName | Cancer-drug dataset |
| --- | --- | --- | --- |
| path:00564_1 | 6 | Glycerophospholipid metabolism | BLCA-Cisplatin |
| path:00564_1 | 6 | Glycerophospholipid metabolism | BLCA-Cisplatin |
| path:04014_1 | 7 | Ras signaling pathway | BLCA-Cisplatin |
| path:04022_1 | 9 | cGMP-PKG signaling pathway | BLCA-Cisplatin |
| path:04068_1 | 9 | FoxO signaling pathway | BLCA-Cisplatin |
| path:04070_1 | 9 | Phosphatidylinositol signaling system | BLCA-Cisplatin |
| path:04310_1 | 6 | Wnt signaling pathway | BLCA-Cisplatin |
| path:04510_1 | 13 | Focal adhesion | BLCA-Cisplatin |
| path:00565_1 | 7 | Ether lipid metabolism | BLCA-Gemcitabine |
| path:04012_1 | 5 | ErbB signaling pathway | BLCA-Gemcitabine |
| path:04015_1 | 6 | Rap1 signaling pathway | BLCA-Gemcitabine |
| path:04020_1 | 17 | Calcium signaling pathway | BLCA-Gemcitabine |
| path:04022_2 | 10 | cGMP-PKG signaling pathway | BLCA-Gemcitabine |
| path:04062_1 | 6 | Chemokine signaling pathway | BLCA-Gemcitabine |
| path:04151_1 | 11 | PI3K-Akt signaling pathway | BLCA-Gemcitabine |
| path:04152_1 | 9 | AMPK signaling pathway | BLCA-Gemcitabine |
| path:04510_2 | 10 | Focal adhesion | BLCA-Gemcitabine |
| path:04630_1 | 8 | Jak-STAT signaling pathway | BLCA-Gemcitabine |
| path:04662_1 | 6 | B cell receptor signaling pathway | BLCA-Gemcitabine |
| path:04722_1 | 6 | Neurotrophin signaling pathway | BLCA-Gemcitabine |
| path:04910_1 | 10 | Insulin signaling pathway | BLCA-Gemcitabine |
| path:04917_1 | 8 | Prolactin signaling pathway | BLCA-Gemcitabine |
| path:05200_1 | 6 | Pathways in cancer | BLCA-Gemcitabine |
| path:05214_1 | 6 | Glioma | BLCA-Gemcitabine |
| path:05215_1 | 5 | Prostate cancer | BLCA-Gemcitabine |
| path:05220_1 | 8 | Chronic myeloid leukemia | BLCA-Gemcitabine |
| path:00480_1 | 10 | Glutathione metabolism | LGG-Temozolomide |
| path:00500_1 | 5 | Starch and sucrose metabolism | LGG-Temozolomide |
| path:00980_1 | 4 | Metabolism of xenobiotics by cytochrome P450 | LGG-Temozolomide |
| path:04062_2 | 9 | Chemokine signaling pathway | LGG-Temozolomide |
| path:04310_2 | 8 | Wnt signaling pathway | LGG-Temozolomide |
| path:04630_2 | 9 | Jak-STAT signaling pathway | LGG-Temozolomide |
| path:04670_1 | 8 | Leukocyte transendothelial migration | LGG-Temozolomide |
| path:04713_1 | 7 | Circadian entrainment | LGG-Temozolomide |
| path:04723_1 | 7 | Retrograde endocannabinoid signaling | LGG-Temozolomide |
| path:04724_1 | 10 | Glutamatergic synapse | LGG-Temozolomide |
| path:04725_1 | 11 | Cholinergic synapse | LGG-Temozolomide |
| path:04726_1 | 6 | Serotonergic synapse | LGG-Temozolomide |
| path:04727_1 | 7 | GABAergic synapse | LGG-Temozolomide |
| path:05032_1 | 7 | Morphine addiction | LGG-Temozolomide |
| path:05200_1 | 6 | Pathways in cancer | LGG-Temozolomide |
| path:04151_2 | 12 | PI3K-Akt signaling pathway | PAAD-Gemcitabine |
| path:04510_3 | 15 | Focal adhesion | PAAD-Gemcitabine |
| path:00240_1 | 13 | Pyrimidine metabolism | STAD-Fluorouracil |
| path:04010_1 | 8 | MAPK signaling pathway | STAD-Fluorouracil |
| path:04068_2 | 15 | FoxO signaling pathway | STAD-Fluorouracil |
| path:04152_2 | 9 | AMPK signaling pathway | STAD-Fluorouracil |
| Total |  | 46 | 5 |
