# Supplementary figures and images for: Identifying subpathway signatures for individualized anticancer drug response by integrating multi-omics data
Source: J Transl Med. 2019 Aug 6;17:255. doi: 10.1186/s12967-019-2010-4 (PMC6685260; doi:10.1186/s12967-019-2010-4)

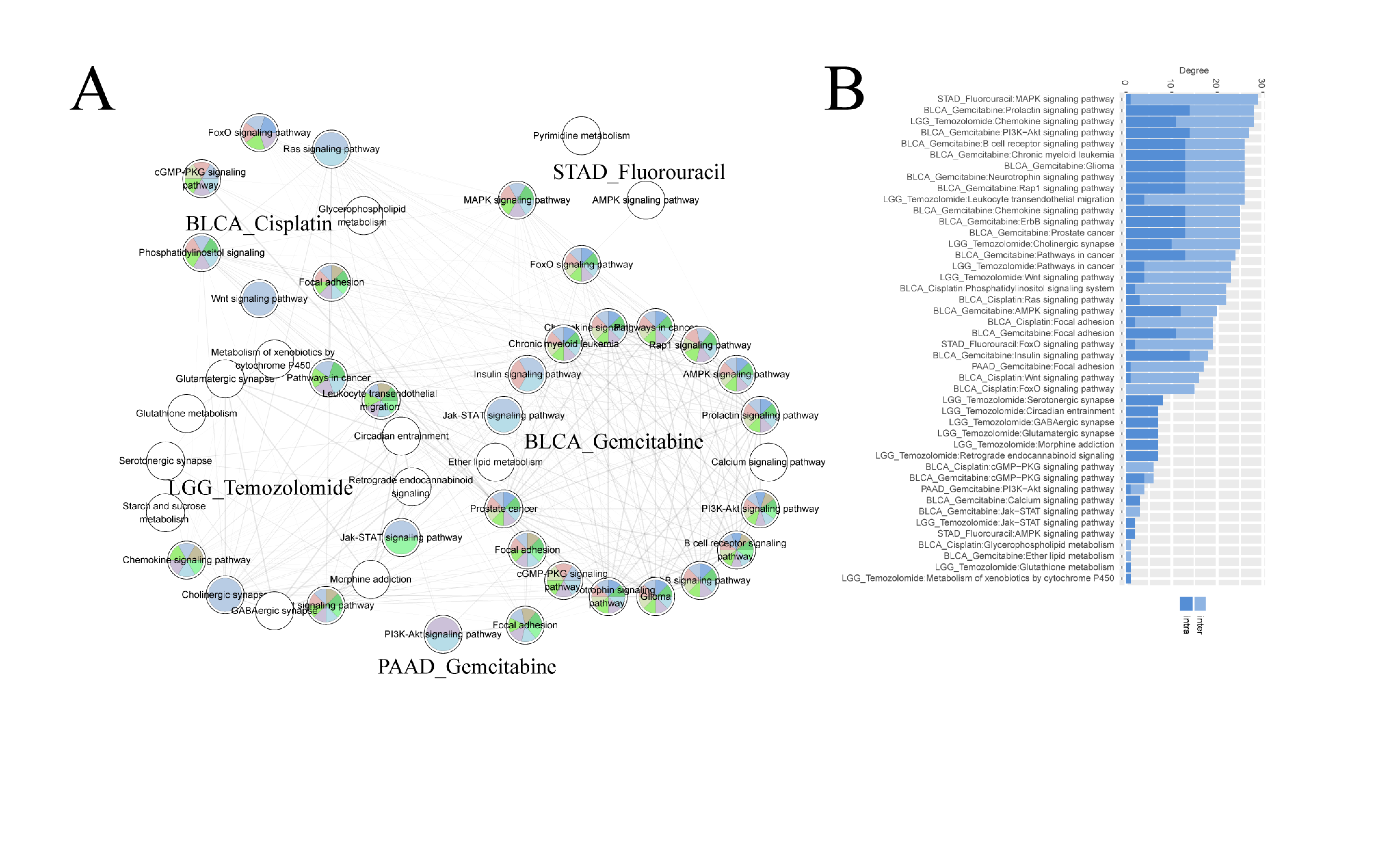

Supplement: Supplementary file 4 — Additional file 4: Figure S2. Functional connections between subpathway signatures associated with the responses of different drugs. (A) Functional similarity network of subpathway signatures identified for different anticancer drugs in various tumor types. Node indicates subpathway and edge indicates the semantic similarity score greater than 0.6 from GOSemSim between two subpatwhays. Pie chart in the node indicates pathway related cancer hallmarks. (B) The degree distribution of nodes in (A). Dark blue represents the number of intra connections between subpathway signatures from the same dataset. Light blue indicates the number of inter connections between subpathway signatures from different datasets. [file 12967_2019_2010_MOESM4_ESM.tif]

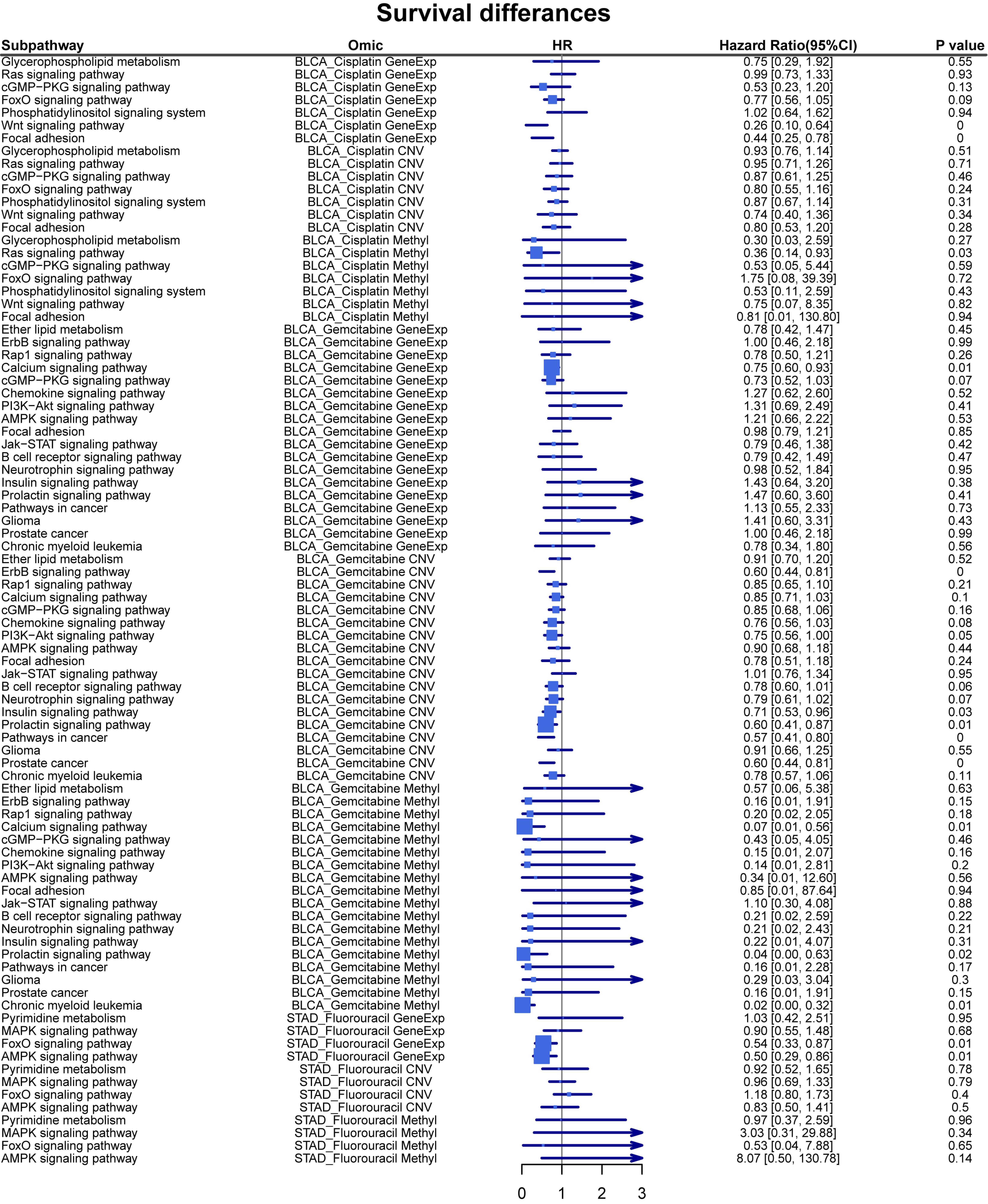

Supplement: Supplementary file 5 — Additional file 5: Figure S3. Discriminative prognosis power of the identified subpathway signatures. (A) Forest plot indicates HRs and 95% CI from univariate Cox proportional hazards model of the subpathway signatures at three omic level from BLCA-Cisplatin, BLCA-Gemcitabine and STAD-Fluorouracil. [file 12967_2019_2010_MOESM5_ESM.tif]

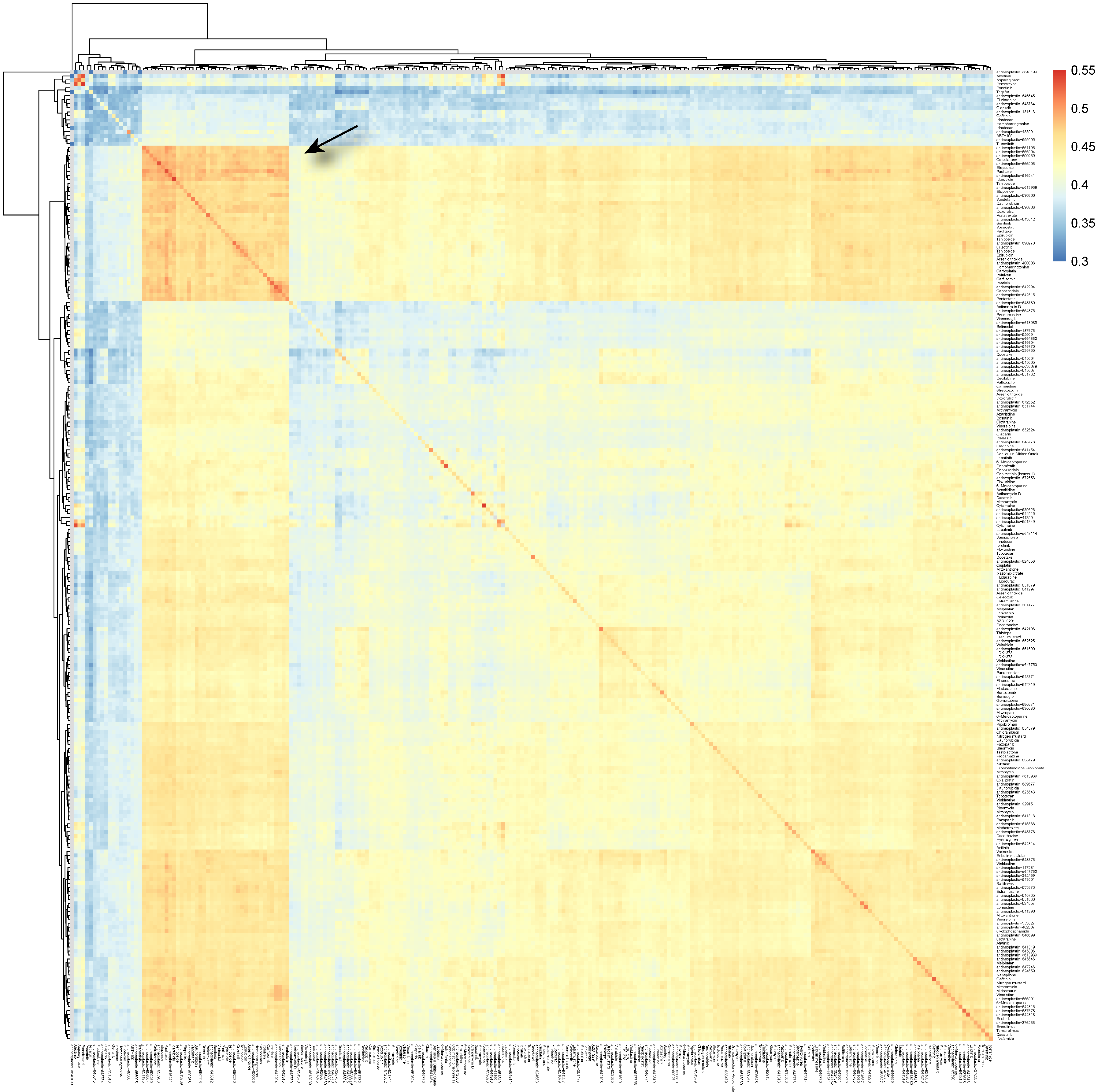

Supplement: Supplementary file 6 — Additional file 6: Figure S4. Unsupervised hierarchical clustering of drugs based on the mean semantic similarity scores of subpathways related with their responses. [file 12967_2019_2010_MOESM6_ESM.tif]
